# Very Low-Dose Sublingual Ketamine for Borderline Personality Disorder and Treatment-Resistant Depression

**DOI:** 10.7759/cureus.57654

**Published:** 2024-04-05

**Authors:** Mitchell Liester, Rachel Wilkenson, Barry Patterson, Bertrand Liang

**Affiliations:** 1 Department of Psychiatry, University of Colorado School of Medicine, Aurora, USA; 2 Department of Psychiatry, Matthews-Vu Medical Group, Colorado Springs, USA; 3 Department of Pharmacy, The Medicine Shoppe, Colorado Springs, USA; 4 Department of Neurology, University of Colorado School of Medicine, Aurora, USA

**Keywords:** glutamate dysregulation, neuroplasticity enhancement, ampa receptor, brain-derived neurotrophic factor, borderline personality disorder (bpd), treatment resistant depression (trd), low dose ketamine

## Abstract

Borderline personality disorder (BPD) and treatment-resistant depression (TRD) are common mental disorders that are challenging to treat. Ketamine is an N-methyl-D-aspartate receptor antagonist that has shown promise as a rapid-acting antidepressant when administered intravenously. BPD symptoms have also been demonstrated to improve with repeated intravenous administration of ketamine, and a single case report described improvement in BPD following the intranasal administration of esketamine. We present a case report of a woman with BPD and TRD who responded to treatment with very low-dose sublingual ketamine. Very low-dose sublingual ketamine may be effective for the treatment of psychiatric disorders such as BPD and/or comorbid TRD.

## Introduction

Borderline personality disorder (BPD) is a common mental disorder associated with severe functional impairment, high rates of comorbid mental disorders, intensive use of treatment, high costs to society, and a high rate of suicide. The median prevalence of BPD is estimated to be 1.35%, and the rate of having one or more comorbid psychiatric disorders is 84.5%. The mortality rate from suicide is between 8% and 10% [1]. Currently, no medication has been approved for the treatment of BPD.

The biopsychosocial model of BPD posits that this disorder results from a combination of genetic factors and adverse childhood experiences, resulting in affective and behavioral dysregulation as well as disturbed relatedness. Excitotoxicity resulting from dysregulated glutamate signaling has also been suggested to play a role in the pathophysiology of BPD [1]. Currently, no medication has been approved for the treatment of BPD.

Definitions of treatment-resistant depression (TRD) vary. A commonly employed definition is depression that is resistant or refractory when at least two trials of antidepressants from different pharmacologic classes (which are adequate in terms of dosage, duration, and compliance) fail to produce a significant clinical improvement. TRD is relatively common in clinical practice, with 35% of patients failing to achieve an adequate response following treatment with two antidepressants [2].

The etiology of TRD is likely multifactorial. Potential contributing factors include dysregulation of the hypothalamic-pituitary-adrenal axis, genetic polymorphisms of receptor and transporter genes [3], and neuroinflammation [4]. Glutamate dysregulation has been suggested to play a role in the etiology of TRD, and treatments that modulate glutamate transmission have been proposed as treatments for TRD [5]. One of these glutamatergic regulators is ketamine.

Ketamine is an N-methyl-D-aspartate receptor antagonist that was first synthesized in the 1960s [6]. In 1970, ketamine was approved by the US FDA for use as a general anesthetic [7].

At high doses, ketamine exerts neurotoxic effects through a process known as excitotoxicity. However, at lower (i.e., subanesthetic) doses, ketamine exhibits neuroprotective effects, which are believed to be mediated by the release of the neurotrophin brain-derived neurotrophic factor (BDNF) [8]. Even low doses of ketamine can cause side effects. The most commonly reported side effects of low-dose sublingual troches are dizziness, sedation, and impaired cognitive functioning. However, if these side effects occur, they are generally mild and transient (i.e., five to 60 minutes).

A low dose (0.5 mg/kg) of ketamine administered intravenously (IV) produces significant improvement in depression within 72 hours [9]. An even lower dose (10 mg) administered sublingually (SL) and repeated every two to seven days produces rapid and sustained improvement in mood, cognition, and sleep in individuals with TRD [10]. Ketamine also shows promise for reducing suicidal ideation [11].

A recent randomized control trial evaluated the effects of a single dose of IV ketamine in individuals with BPD. The treatment group, which consisted of 10 adults with BPD, was administered a single dose (0.5 mg/kg) of ketamine IV, and the control group (n = 12) received a single IV dose (0.04 mg/kg) of midazolam. The investigators found a nonstatistically significant reduction in suicidal ideation and depression in the treatment group vs. the control group as measured by the Beck Scale for Suicide Ideation and the Beck Depression Inventory (BDI) [11].

A second study involved a retrospective analysis of data collected from participants who received care at the Canadian Rapid Treatment Center of Excellence [12]. The investigators evaluated the effectiveness of IV ketamine in 100 individuals with TRD and comorbid BPD, as well as in 50 individuals with TRD but no BPD. Participants received four individual doses of IV ketamine (0.5-0.75 mg/kg) over a two-week time period. The primary outcome measures were changes in depressive symptom severity and borderline symptom severity as measured by the Quick Inventory of Depressive Symptomatology (QIDS SR-16) and Borderline Symptom List 23 (BSL-23). Both the BPD-positive group and the BPD-negative group exhibited a significant reduction in symptoms of depression, as indicated by changes in their QIDS SR-16 scores. However, there were no statistically significant between-group differences in the response rates. In the BPD-positive group, there was a significant correlation between the change in the BSL-23 score and the change in the QIDS SR-16 total score (p < 0.0001). A reduction in BPD severity was also found, with 34.3% of the BPD-positive group exhibiting a response and an additional 22.9% exhibiting a partial response to treatment, as indicated by a reduction in BSL-23 scores. Suicidality was reduced in both the BPD-positive and the BPD-negative groups, as indicated by a reduction in the QIDS SR-16 suicide item score [12].

Case reports also describe improvements in depression in patients with BPD. Nandan et al. [13] described a patient with BPD whose depression improved following treatment with intranasal esketamine, as measured by a gradual reduction in Hamilton Depression Rating Scale scores over a two-year period. Rogg et al. [14] reported improvement in both depressive symptoms and BPD symptoms in a patient who received IV ketamine infusions. Improvement was demonstrated by a reduction in BDI-II and BSL-23 scores.

The bioavailability of SL ketamine is approximately 29% [15]. SL administration thus results in significantly lower blood levels than an equivalent IV dose. This lower dose, combined with ease of administration and a relatively low cost (approximately US$15-30/month), renders this route of administration a convenient option for both patients and physicians. However, because sublingual ketamine troches are not produced by pharmaceutical companies, they must be prepared by a compounding pharmacy.

The finding that low-dose SL ketamine improves depressive symptoms in patients with TRD and the discovery that patients with BPD improve following treatment with IV ketamine led to the decision to utilize low-dose SL ketamine as a treatment for a patient with TRD and BPD.

## Case presentation

The patient was a 60-year-old female who presented to the clinic in March 2015 with a 34-year history of BPD, recurrent depression, daily suicidal ideation, generalized anxiety disorder, and panic disorder. She first sought psychiatric treatment in 1990 following a motor vehicle accident (MVA) in which she sustained a whiplash injury. She was subsequently diagnosed with post-traumatic stress disorder due to the trauma of the MVA. The patient also reported a history of verbal and physical abuse by her mother when she was young.

At her initial visit, the patient’s medications were imipramine 150 mg daily at bedtime, risperidone 0.5 mg at bedtime, quetiapine 100 mg at bedtime as needed for sleep, and lorazepam 1 mg daily as needed for anxiety. Previous medications tried and discontinued due to an inadequate therapeutic response included aripiprazole, bupropion, buspirone, citalopram, divalproex sodium, eszopiclone, fluoxetine, L-methylfolate, lamotrigine, lorazepam, risperidone, sertraline, trazodone, zaleplon, and zolpidem. The patient had also worked with several psychotherapists over a period of multiple years.

The patient’s family history was notable for both anxiety and depression. She was admitted to inpatient psychiatric facilities four times and attempted suicide twice. Her first two psychiatric hospitalizations were in 1993 and 2005. Her first suicide attempt occurred in 2011 when she overdosed on her medication. She was subsequently admitted to a psychiatric hospital for two days. Her second suicide attempt occurred in 2016 when she walked in front of a semi-truck on an interstate highway. Fortunately, she survived this suicide attempt, although she sustained multiple injuries, including a fractured left arm, three fractured ribs, and multiple facial fractures. She also suffered a traumatic brain injury. The patient was admitted to the intensive care unit of a general medical hospital and remained hospitalized for five days before being transferred to a psychiatric hospital, where she received six days of inpatient psychiatric treatment. Following this suicide attempt, the patient felt disappointed that she had survived.

In October 2022, the patient was evaluated and reported ongoing symptoms of depression, including depressed mood (rated as 3 on a 0-10 scale), difficulty falling and staying asleep, fatigue, 20-pound weight loss, impaired concentration, moderate anxiety, anhedonia, and daily suicidal ideation. Her medications at that time included imipramine 50 mg daily, lamotrigine 200 mg daily, eszopiclone 3 mg at bedtime as needed for sleep, and quetiapine 50-200 mg at bedtime as needed for sleep. The patient’s scores were 33 on the Montgomery-Åsberg Depression Rating Scale (MADRS) (moderate depression) and 21 on the Patient Health Questionnaire-9 (PHQ-9) (severe depression). Due to numerous failed medication trials and persistent symptoms of depression, the patient was prescribed sublingual ketamine troches with directions to take 25 mg every third day for four doses, then 25 mg every other day for four doses, then 25 mg daily. This medicine was taken at bedtime. She did not initiate the ketamine troches until the end of December 2022.

The patient was seen for a follow-up three months after initiating treatment with ketamine. She described feeling more positive about her marriage, improving emotional regulation, and having stopped raging. She described increased resilience as indicated by her improved ability to manage stressors. The patient had begun talking with her oldest daughter and seeing her grandchildren for the first time in two years. Previously, the patient’s daughter had refused any contact between her mother and herself or her children due to her mother’s emotional instability. The patient also reported that her fibromyalgia pain was nearly gone, a change she related to an improved ability to manage her emotions. Regarding her symptoms of depression, the patient reported her mood was “happier,” and she rated her mood as 7-8 on a 0-10 scale, although no formal rating scales were used at that time. Her sleep had improved, and she was averaging seven hours of sleep per night. Her energy level was “much better.” She described an improved appetite, and she had regained 10 pounds that she had lost previously. Her concentration improved, and her anxiety diminished. She denied anhedonia. She was no longer experiencing suicidal ideation.

After 10 months of treatment with 25 mg of sublingual ketamine daily, the patient showed sustained improvement. She had begun working a second job. Her mood was euthymic, and she rated her mood as 8/10. She was sleeping well. Her energy level was “good.” Her appetite stabilized. She was able to concentrate well, and her anxiety level remained low. She denied anhedonia. Suicidal ideation remained in remission. Her fibromyalgia pain was significantly reduced relative to her pre-ketamine level. She described an improved ability to manage stressors in her life. She denied adverse effects related to her medicines, such as dizziness or sedation. The patient’s PHQ-9 score was 7, placing her in the range of mild depression, and her MADRS score was 1, indicating no depression.

The patient’s BPD symptoms were also markedly improved. She no longer feared abandonment, and her relationships had deepened. Her relationship with her older daughter and grandchildren remained connected, and her daughter now sought her out for advice. Her self-image had improved, and she no longer experienced impulsivity. She was working two jobs and enjoying both of them. She denied suicidal ideation or thoughts of self-harm. Her affective instability had resolved, and she was no longer experiencing episodes of anxiety or rage. She was not experiencing paranoia or severe dissociative symptoms. The patient no longer met the Diagnostic and Statistical Manual of Mental Disorders (DSM-V) diagnostic criteria for BPD.

## Discussion

Ketamine’s antidepressant mechanism of action remains to be elucidated, but the neurotransmitter glutamate has been demonstrated to play a key role. Glutamate is a nonessential amino acid and is the primary excitatory neurotransmitter in the central nervous system.

Activation of glutamatergic α-amino-3-hydroxy-5-methyl-4-isoxazolepropionic acid receptors (AMPARs) increases Na+ influx through the AMPAR channels, triggering a release of Ca2+ from intracellular stores [16]. Increased intracellular Ca2+ stimulates the release of BDNF from post-synaptic neurons, which then binds to tropomyosin receptor kinase B, activating the mechanistic target of the rapamycin (mTOR) pathway [17]. Activation of mTOR stimulates the production of proteins that facilitate dendritic arborization, synaptogenesis [18], and neurogenesis [19]. These neuroplastic changes are proposed to repair damage resulting from glutamate dysregulation, which has been implicated in the pathophysiology of TRD and BPD [5,20]. It is hypothesized that daily low-dose sublingual ketamine may facilitate neuroplastic changes that improve BPD and TRD.

There is limited data on the use of lower doses of ketamine in clinical medicine, notwithstanding either BPD or TRD. The doses used in IV preparations for the treatment of depression or BPD typically range from 0.5 mg/kg to 0.75 mg/kg. Such doses would result in a bioavailability of 80-85% higher than would be expected when utilizing a low dose of 25 mg SL. Earlier studies, particularly in psychiatric diseases, rarely evaluated such low doses. The one exception is a study by Lara et al. [10] documenting that a 10 mg dose administered every two to seven days effectively treated patients with TRD.

The case described here suggests lower doses, administered SL, may be helpful in certain clinical phenotypes, particularly those where BPD is predominant with comorbid TRD. Whether this is generalizable to those with either of these disorders alone or other comorbidities and/or other risk factors (e.g., trauma) will require further study. However, this initial case report of a patient with BPD and TRD responding to a very low dose (25 mg) of daily sublingual ketamine suggests lower doses may have effectiveness in some psychiatric disorders. Furthermore, possible advantages of sublingual versus IV ketamine include lower cost, reduced side effects, the convenience of at-home dosing, and the option of daily dosing, which provides more frequent pulsing of BDNF to facilitate neuroplastic changes in the central nervous system.

## Conclusions

BPD is chronic and debilitating, and no medication has yet been approved to treat this disorder. Ketamine was approved by the FDA more than 50 years ago for use as a general anesthetic, and for more than two decades, it has demonstrated potent efficacy as an off-label treatment for depression. This case documents that very low-dose ketamine can be used to address patients with BPD and TRD. If confirmed in a larger series, SL ketamine could be an inexpensive, safe medication added to the armamentarium of those who treat BPD, TRD, and potentially other related psychiatric disorders.
